# Distributional Vowel Training Is Less Effective for Adults than for Infants. A Study Using the Mismatch Response

**DOI:** 10.1371/journal.pone.0109806

**Published:** 2014-10-07

**Authors:** Karin Wanrooij, Paul Boersma, Titia L. van Zuijen

**Affiliations:** 1 Amsterdam Center for Language and Communication, University of Amsterdam, Amsterdam, The Netherlands; 2 Research Institute of Child Development and Education, University of Amsterdam, Amsterdam, The Netherlands; University of Jyväskylä, Finland

## Abstract

Distributional learning of speech sounds (i.e., learning from simple exposure to frequency distributions of speech sounds in the environment) has been observed in the lab repeatedly in both infants and adults. The current study is the first attempt to examine whether the capacity for using the mechanism is different in adults than in infants. To this end, a previous event-related potential study that had shown distributional learning of the English vowel contrast /æ/∼/ε/ in 2-to-3-month old Dutch infants was repeated with Dutch adults. Specifically, the adults were exposed to either a bimodal distribution that suggested the existence of the two vowels (as appropriate in English), or to a unimodal distribution that did not (as appropriate in Dutch). After exposure the participants were tested on their discrimination of a representative [æ] and a representative [ε], in an oddball paradigm for measuring mismatch responses (MMRs). Bimodally trained adults did not have a significantly larger MMR amplitude, and hence did not show significantly better neural discrimination of the test vowels, than unimodally trained adults. A direct comparison between the normalized MMR amplitudes of the adults with those of the previously tested infants showed that within a reasonable range of normalization parameters, the bimodal advantage is reliably smaller in adults than in infants, indicating that distributional learning is a weaker mechanism for learning speech sounds in adults (if it exists in that group at all) than in infants.

## Introduction

“Distributional learning” is learning from simple exposure to the frequency distributions of stimuli in the environment [1], [2]. It is assumed to be an important mechanism by which infants start to learn the phonemes of their native language (e.g., [3]). In the lab, where exposure to speech sound distributions lasts only a few minutes, the mechanism has been reported not only for infants, but also for adults who try to master difficult speech sound contrasts of a second language (Introduction section 1).

Some of the previous research suggests that the capacity for distributional learning of speech sounds is smaller in adults than in infants (Introduction section 2), while other research implies that this capacity remains fairly robust in adulthood (Introduction section 3). Here we present the first attempt to *directly* compare adults and infants in their capacity for distributional learning of speech sounds. For this, a recent distributional learning experiment with infants [4] was repeated with adults, and the effect of distributional training on the adults’ neural auditory discrimination performance was compared to that of the infants.

### 1. Distributional learning

**Figure 1:**
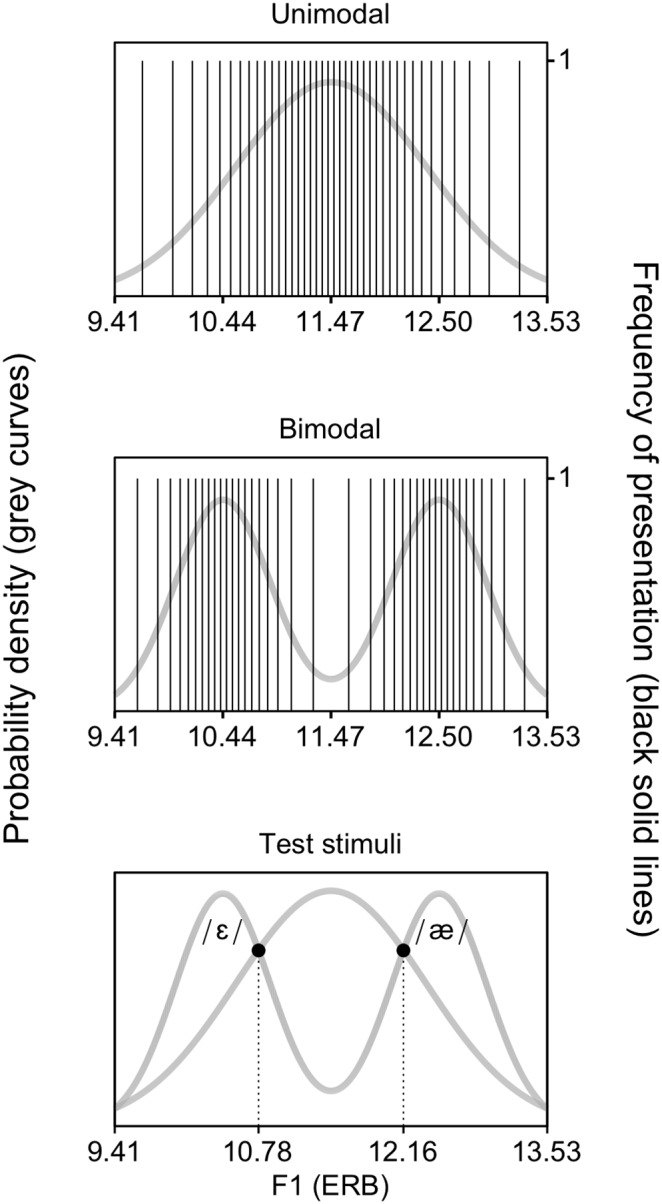
Distributions of first formant (F1) values (in ERB). The unimodal (top) and bimodal (middle) distributions represent the Dutch vowel /ε/ and the English vowel contrast /ε/∼/æ/, respectively. Each solid vertical line indicates a vowel token with a specific F1 value. Each vowel token was presented only once (i.e., the height of the vertical lines is 1). The grey curves are the underlying probability density functions. When creating training distributions, the acoustic values of the test stimuli can be calculated by computing the intersections (black discs, bottom) of the unimodal and bimodal distributions.

The concept of distributional learning can be illustrated best with an example. The chosen example is relevant in the current study, where we use distributions encompassing the same speech sound contrast, namely the English vowel contrast /æ/∼/ε/ as in *bat* vs. *bet*. In Southern British English (SBE) the vowels in this contrast differ primarily in the first and second formants (F1 and F2). Specifically, /æ/ has a higher F1 and a lower F2 than /ε/ [5]. For the sake of clarity, we focus on the F1 values only. When hypothetically measuring the F1 values in many tokens of SBE /æ/ and /ε/ (mixed), it can be observed that the values are grouped around two values, one for the mean of /æ/ and one for the mean of /ε/. This is illustrated in the middle graph of Figure 1. Each vertical line indicates an F1 value. The curve shows the underlying probability density function. Because the function has two peaks, the distribution is called bimodal.

In Dutch the contrast /æ/∼/ε/ is not phonemic, and Dutch listeners show difficulty in mastering it (e.g., [6]–[9]). This is probably because Dutch has the single vowel /ε/ (as in the Dutch word *pet*, “cap”) in roughly the region of the F1-by-F2 vowel space occupied by SBE /æ/∼/ε/ [10], [11]. When hypothetically measuring the F1 values in many tokens of Dutch /ε/, the values cluster around a single value, which is the mean F1 of Dutch /ε/ (top graph of Figure 1). Consequently, the underlying probability density function (the curve) has only one peak and is thus unimodal. Distributional learning reflects the idea that the language-specific distributions cause English listeners to experience two vowels in this region of the vowel space, and Dutch listeners one vowel.

The existence of distributional learning has been demonstrated in the lab, where exposure to speech sound distributions takes just a few minutes. In a typical distributional learning experiment, participants (e.g., the Dutch infants in [4]) are exposed to either a bimodal distribution of speech sounds representing a contrast to be acquired (e.g., the SBE contrast /æ/∼/ε/, as for one group of infants in [4]) or to a unimodal distribution that represents a single native speech sound (e.g., Dutch /ε/, as for a second group of infants in [4]). After exposure, participants are tested on their discrimination or identification of two tokens that were represented equally in both distributions during training (e.g., for the infants in [4]: an [æ] and an [ε], as illustrated by the black discs in the bottom graph of Figure 1). If distributional training is effective, bimodally trained listeners should discriminate or identify the two test stimuli better than unimodally trained listeners, because the bimodal distribution is expected to make listeners experience the test stimuli as belonging to different speech sound categories and the unimodal distribution is expected to make them experience these stimuli as being representatives of a single speech sound category. Indeed, several studies report such an effect of distributional training, both studies with infants (including [4], and [12]–[15]), and studies with adults [16]–[22].

### 2. Previous research with plosive distributions

Only one set of studies has examined distributional learning of the same speech sound contrast in adults [16], [17], [19] *and* infants [12]–[14], namely the voicing contrast between the “voiced” plosive in the English word *day* and a voiceless unaspirated plosive similar to that in the English word *stay*, with participants from English homes. The overall results suggest in a weak manner (namely, by comparing multiple degrees of significance, which does not constitute a valid statistical test) that distributional learning, which was observed in both adults and infants, might have a smaller scope in the former than in the latter group. Specifically, for infants, exposure to a bimodal distribution of the voicing contrast at one place of articulation (e.g., a distribution of [d]∼[t]) turned out to enhance discrimination of the same contrast at *another* place of articulation (e.g., between [g] and [k]) [13], whereas for adults the parallel results were not significant [17]. Also, Yoshida et al. [14] argue that the capability to learn from exposure to a speech sound distribution may weaken with age already within the first year of life. Two groups of 10-to-11-month olds in this study did not improve discrimination significantly after a 2.3-minute bimodal training (which is the same duration as used earlier for the younger infants, who were reported to exhibit distributional learning [12], [13]). After a longer training (4.6 minutes) an additional group of 10-to-11-month olds did exhibit significantly improved discrimination (a direct comparison between the three groups was not reported). Exposure duration in the adult studies [16], [17], [19] was chosen to be even longer (9 minutes).

In sum, on the basis of this set of studies (i.e., those using plosive distributions), one might hypothesize that distributional learning is a less prominent mechanism in adults than in infants. Unfortunately, the method differed between the adult and infant studies in several aspects (including the actual stimuli, the procedure and, as just mentioned, the training duration). Moreover, as said above, neither adults and infants, nor older infants and younger infants, nor groups exposed to different training durations, were compared with a direct statistical test. Consequently, the studies in this set cannot really be interpreted as providing evidence for a declining prominence of distributional learning with age. Also, the contrast used in this set was a voicing distinction in plosives, for which the distributional learning mechanism may be very different from the distributional learning of vowels, which we investigate in the current study (Introduction section 4).

### 3. Previous research with vowel distributions

A second set of studies on distributional learning used vowel distributions, as we do in the present study, and also includes both studies with adults [18], [20]–[22] and a study with infants [4]. The results demonstrate that an effect of distributional training *can* be measured in adults after short exposure (5 minutes in [18], less than 2 minutes in [20]–[22]), thus suggesting that the capacity for distributional learning can remain rather robust in adulthood. Unfortunately, the vowel contrasts used for the adults (Dutch /ɑ/~/a:/ and /ɪ/~/i/ for Bulgarian learners [18]; Dutch /ɑ/~/a:/ for Spanish learners [20]–[22]) do not match those for the infants (SBE /æ/∼/ε/ for Dutch infants [4]), and test procedures differed between the adult and infant studies. Consequently, it is not clear how the observed effects of distributional training in adults relate to those in infants.

### 4. The objective of the current study

As explained above (Introduction sections 2 and 3), previous research implies conflicting conclusions about the capacity for distributional learning in adults as compared to that in infants. On the one hand, this capacity may decline with age (Introduction section 2). On the other hand, the capacity for distributional learning seems robust regardless of age, as it is measurable in a fast distributional training paradigm in both infancy and adulthood (Introduction section 3). The purpose of the current study was to shed light on the effect of age on the capacity for distributional learning. Specifically, the aim was to directly compare adults’ capacity for distributional learning to that of infants, and thus to determine the relative importance of the mechanism for learning speech sounds in adulthood, when speech sounds of new languages are learned, versus that in infancy, when the speech sounds of the native language are learned. In order to examine whether adults have a smaller capacity for distributional learning than infants, we first repeated a recent study that demonstrated an effect of distributional training of SBE /æ/∼/ε/ in Dutch infants aged 2 to 3 months ([4]), with Dutch adults. Subsequently, we aimed to determine whether any observed effect of distributional training in the adults was smaller than the corresponding effect observed in the infants in [4].

### 5. Comparing distributional learning in infants and adults

In any comparison between participant groups, it is important to use the same method, i.e., the exact same training, with the same duration, and the same method for testing discrimination after training for all participant groups. A method that can be used for both infants and adults to test discrimination after distributional training is the measurement of the mismatch response (MMR), a brain response that can be calculated from event-related potentials (ERPs). The MMR has been related to behavioral discrimination in adults (for a review see [23]) and has been used widely to test discrimination in newborns (e.g., [24], [25]), older infants (e.g., [26], [27]), children (e.g., [28], [29]) and adults (e.g., [30], [31]). The MMR has also been used to compare speech sound discrimination in infants versus adults [32].

The MMR can be recorded in an oddball paradigm [33], in which infrequent “deviant” stimuli (e.g., [æ]) appear randomly in a train of “standard” stimuli (e.g., [ε] tokens). If the auditory system signals a difference between the standards and the deviants, it will generate different brain responses (ERPs) to the two kinds of stimuli. This difference between the ERP to the deviants and that to the standards is the MMR. Larger perceived differences between standard and deviant stimuli have been related to larger MMR amplitudes, not only in adults [30], [34], but also in children [28] and in one-year old infants [35].

The cause of the MMR method being suitable for infants and adults alike is that the MMR reflects automatic auditory processing, which occurs before participants can pay conscious attention to the stimuli [36], and which is elicited even if participants do not attend to the stimuli at all [33], [37], [38]. Consequently, the response does not depend on a behavioral task, which young infants cannot perform. The MMR thus allows for minimizing methodological differences between testing infants and testing adults on their discrimination performance.

When comparing the MMR of infants and adults, a point of concern is that the infant and adult MMR may not reflect the same neural processes: the underlying ERPs have a very different morphology in infants than in adults, which is probably partly due to structural differences (i.e., the size and anatomical structure of the brain and skull), and partly to representational differences (i.e., linguistic representations are likely to be either absent or immature in infants). Notice, however, that as the MMR is a difference wave (see above in this section), part of the differences between infant and adult ERPs is removed by the subtraction. Nevertheless, in order to compensate for differences between infant and adult MMRs that cannot be avoided by using the same method and by subtracting ERPs, some kind of normalization has to be performed that scales the MMR amplitudes prior to statistical analysis (Method section 7). Normalization between infant and adult MMRs was applied before ([32]), albeit without a specification of the exact normalization method.

In order to facilitate the comparison of the effect of distributional training between infants and adults, the present study: (1) minimizes methodological differences by measuring the MMR in adults, as was done for the infants in [4], and (2) normalizes the MMR amplitudes before statistical analysis.

In sum, the present study first examines whether distributional training of SBE /æ/∼/ε/ is effective for Dutch adults, by repeating an experiment that demonstrated an effect of such training in Dutch 2-to-3-month-old infants [4]. Specifically, we expose the Dutch adults to either a bimodal or a unimodal distribution encompassing /æ/∼/ε/, and then test their discrimination of a representative [ε] and [æ] by recording the MMR in an oddball paradigm. On the basis of earlier reported effects of distributional training in adults (discussed in Introduction sections 2 and 3), it is expected that the bimodally trained participants will discriminate the test vowels better, and will thus have a larger MMR amplitude, than the unimodally trained listeners. Secondly, we examine whether the difference in the normalized MMR amplitude between bimodally and unimodally trained participants is indeed smaller in the adults than in the infants in [4].

## Method

Below we first describe the method for determining whether distributional vowel training is effective for Dutch adults. This method is identical to that used in the previous infant study [4], except where stated otherwise. The final section (Method section 7) explains our approach to normalizing the MMR amplitudes across infants and adults.

### 1. Design

All adults received a pre-test, a training and a post-test. Because the infants in [4] did not do a pre-test, the pre-test data will not be discussed in this paper. The reason for not doing a pre-test with infants was that such a test could be an additional distributional training that distorts the intended training distributions ([4]: 9); since there is strong evidence that adults do not learn in “passive” tests (i.e., where they do not have to perform a specific task and can ignore the presented stimuli, as was the case in the present experiment; e.g., [39]), a pre-test was included for the adults to permit later comparisons with other studies on distributional training of adults [18], [20]–[22].

During training, participants listened to either a unimodal or a bimodal distribution of vowels encompassing /æ/∼/ε/ (Method section 4). Distribution Type (unimodal vs. bimodal) was included as the main between-subject factor in the statistical analysis.

In the post-test, the MMR was recorded in an oddball paradigm [33] to assess discrimination of a representative [æ] and [ε] (Method section 4). Half of the participants in each training group listened to standard [æ] and deviant [ε] in the test, and the other half to standard [ε] and deviant [æ]. This was done in view of possible asymmetries in participants’ perception. For instance, an asymmetry predicted by Polka and Bohn [40], [41] can make discrimination easier if relatively central vowels (in the two-dimensional vowel space defined by F1 and F2; e.g., [ε] as compared to [æ]) are presented *before* relatively peripheral vowels (e.g., [æ] as compared to [ε]) than if they are presented in the reverse order. Conversely, an asymmetry predicted on the basis of the “featurally underspecified lexicon” theory by Lahiri and Reetz [42] can make discrimination easier if a vowel specified for the phonological feature [low] (i.e. /æ/) is followed by a vowel not specified for that feature (i.e. /ε/), than if they are presented in the reverse order. To control for such potential biases, Standard Vowel ([ε] vs. [æ]) was included as a between-subject factor in the statistical analysis.

Thus, the statistical analysis of the effect of distributional training on adults’ discrimination performance had the MMR amplitude as the dependent variable, and Distribution Type (unimodal vs. bimodal) and Standard Vowel ([æ] vs. [ε]) as between-subject factors.

### 2. Participants

Participants were native speakers of Dutch that had been raised monolingually, had not lived abroad during childhood, and had never passed more than four weeks in countries where English is the national language. Forty-four participants were tested, of whom 5 were excluded from analysis (see Method section 5). On the basis of the factors Distribution Type and Standard Vowel (Method section 1), the remaining 39 participants belonged to one of four “groups”, namely Unimodal [æ] (n = 9), Unimodal [ε] (n = 10), Bimodal [æ] (n = 9) or Bimodal [ε] (n = 11). Apart from balancing the sexes (there were 2 or 3 men in each of these groups), the assignment to these groups was random. The Unimodal group thus contained 19 participants (mean age 22 years, range 18 to 28 years) and the Bimodal group 20 participants (mean age 22 years, range 18 to 30 years). In the infant study [4], the relevant analysis had been based on a smaller number of participants, namely 11 infants in the Unimodal and Bimodal groups each.

### 3. Ethics statement

The Ethical Committee of the Faculty of Social and Behavioral Sciences at the University of Amsterdam approved the study protocol. Participants were recruited via posters and flyers distributed at the University of Amsterdam and at public places in Amsterdam. Each participant received an information brochure before coming to the lab. The participant signed an informed consent form before the experiment and was paid 20 euros.

### 4. Stimuli and procedure

The stimuli used in the training and in the test were created with the Klatt synthesizer in Praat [43]. All had the same duration (100 ms, including a rise and fall time of 5 ms), fundamental frequency (F0) contour (150 to 112.5 Hz), intensity (70 dB) and third through tenth formants (F3 = 2400 Hz, F4 = 3400 Hz, F5 = 4050 Hz, F6 through F10: previous formant plus 1000 Hz). The stimuli varied in F1 and F2 (see below). All stimuli were played at 70 dB SPL, measured at about one meter from two loudspeakers, where the participant was sitting. The inter-stimulus interval in the training and the tests was 707 ms. Total experimental time was 45.7 minutes (i.e., 12.1 minutes for the training and 16.8 minutes for each test).

#### Training

The unimodal (Figure 1, top) and bimodal (Figure 1, middle) training distributions each consisted of 900 acoustically different vowels, of which the values of the varying parameters (F1 and F2) reflected a probability density function that approximated a continuous distribution. The distributions were made as described in [22]. Both distributions had identical ranges of F1 and F2 values, based on values reported in [5]: 9.41 to 13.53 ERB (Equivalent Rectangular Bandwidth) for F1 and 21.05 to 18.31 ERB for F2 (for details see [4]). The 1800 F1 and F2 values were calculated on the basis of these defined ranges for F1 and F2 and the defined shapes of the distributions, which were based on earlier distributional learning studies (see [4] for details). The unimodal and bimodal mean F1 and F2 values, i.e., the values represented by the peaks of the Gaussian curves in Figure 1, were 11.47 and 19.68 ERB respectively for the unimodal mean, 10.44 and 20.37 ERB for the bimodal mean representing /ε/, and 12.50 and 18.99 ERB for the bimodal mean representing /æ/. The presentation of the stimuli was randomized per listener. Participants were instructed to relax and listen to the vowels carefully. Because the exposure time was longer than in previous behavioral studies on adult distributional learning (namely more than 12 minutes versus 9 minutes in [16], [17], [19], 5 minutes in [18] and less than 2 minutes in [20]–[22]), there was the risk that participants would fail to pay attention to the vowels during the whole training. This had to be avoided because there is extensive evidence that in contrast to infant listeners, adult listeners do not learn if they do not pay attention to the task [39]. Therefore, in order to help participants to keep their attention on the training vowels, they were not only asked to listen carefully, but also to indicate after training how many different vowels they had perceived. The inclusion of a task to keep participants’ attention to the training vowels is not uncommon in studies on adult distributional training [16], [19].

#### Test

The F1 and F2 values of the standard stimulus and the deviant stimulus in the post-test were defined by the intersections of the unimodal and bimodal F1 and F2 distributions (the black discs in Figure 1, bottom). These intersections represent the values that have been trained equally intensively in both distributions. The F1 and F2 values were 10.78 and 20.14 ERB respectively for the stimulus representative of [ε] and 12.16 and 19.22 ERB respectively for the stimulus representative of [æ]. Half of the participants heard [ε] as the standard and [æ] as the deviant, and the other half heard the opposite pattern. The post-test contained 1100 standard tokens and 150 (i.e., 12%) deviant tokens, which is half the numbers presented to the infants in [4]. This was done because we expected less noisy data for the adults. Besides the constraint that minimally three standards (ten at the start of the test) had to appear before each deviant, the presentation of standards and deviants was randomized per participant. Participants watched a silent movie during recording.

### 5. ERP recording and analysis

The ERP recording and analysis were similar to those in [4]. The EEG was recorded with a 64-channel Biosemi Active Two system (Biosemi Instrumentation BV, Amsterdam, The Netherlands). In addition to the 64 electrodes in the cap, reference electrodes were placed on the mastoid processes and the nose. (The nose reference was not used. It was recorded to permit later comparisons with studies that use the nose as a reference). Also, one electrode was placed to the left of the left eye and one to the right of the right eye in order to track horizontal eye movements, and two electrodes were placed above and below the right eye respectively to monitor vertical eye movements. The sampling rate was 8 kHz, which was downsampled to 512 Hz after recording (Biosemi Decimator 86). The subsequent analyses were performed in Praat [43]. The EEG in each channel was referenced to the mastoids (i.e., the mean of the two mastoid signals was subtracted from each of the 64 channel signals), detrended (i.e., a straight line was subtracted from each channel signal in such a way that its beginning and end became zero) and filtered with a zero-phase pass-band filter between 1 and 25 Hz (implemented in the frequency domain; Hann-shaped smoothing 0.5 Hz at the low edge, 12.5 Hz at the high edge). We then extracted from the EEG signal a large number of 500-ms epochs, namely one for each stimulus token. Each epoch started 110 ms before the onset of the stimulus and ended 390 ms after it. Subsequently, we performed a baseline correction on each epoch by subtracting from each of its channels the mean in that channel of the 110 ms before the onset of the stimulus. Subsequently, we removed all epochs that contained a voltage below −75 µV or above +75 µV in one or more of its channels. In this way, we obtained a set of standard epochs and a set of deviant epochs; if the number of deviant epochs was below 100 for a certain participant, we excluded all of this participant’s data from further analysis (this happened for five of the 44 participants).

The data of each remaining participant was simplified in the following way. By averaging over all (at most 1100) standard epochs, we computed the participant’s “mean standard ERP”, which is a 500-ms 64-channel ERP whose average over the first 110 ms is 0. Similarly, we computed the participant’s “mean deviant ERP” by averaging over all (100 to 150) deviant epochs. Finally, we computed the participant’s 64-channel MMR waveform by subtracting the mean standard ERP from the mean deviant ERP.

In this way, ERPs were recorded and analyzed similarly to those of the Dutch infants in [4]. The differences, which reflect adaptations to the measurement of adult as opposed to infant MMRs, were a larger number of electrodes (64 vs. 32), shorter epochs (500 ms vs. 760 ms; see Method section 6) and more stringent norms for artefact rejection (±75 µV vs. ±150 µV) and for the minimum number of deviants (100 vs. 75).

### 6. MMR analysis

Numerous studies have established the *adult* MMR as a negativity (as reflected in the name “mismatch negativity” or MMN; [37]) occurring predominantly at fronto(central) electrodes (when the chosen reference is the nose or the mastoids) in a time frame between roughly 150 and 250 ms after change onset (for a review, see [23]). In many studies, the analysis is confined to the midline frontal electrode Fz (e.g., [30], [31], [44]), because the MMN tends to be prominent there [23].

In line with these properties of the MMN, we performed the following steps for each of the four groups, i.e. for each combination of Distribution Type (i.e., uni- and bimodal) and Standard Vowel ([æ] and [ε]). We first determined the group’s 64-channel waveform by averaging the MMR waveforms of the group’s participants, and then determined the “group latency” as the time of the most negative voltage occurring in this average waveform in the Fz channel between 150 and 250 ms after stimulus onset. Then, we defined a 50-ms “group window” of analysis, starting 25 ms before and ending 25 ms after the group latency. Subsequently, we determined each participant’s “MMR amplitude” at Fz by time-averaging the participant’s MMR waveform at Fz over this window. In this way we reduced the MMR waveform for each participant to one relevant number only.

It should be noted that for the infants in [4] the MMR amplitude had been computed somewhat differently due to the larger uncertainty about the location on the scalp and the timing of the MMR for infants than for adults (for a discussion, see [4]). Because of the uncertainty as to scalp location, the infant response was not analyzed at Fz only, but at eight different electrodes, ranging in scalp position from frontal to central and temporal (parietal and occipital electrodes were not used because several infants had been lying on these electrodes), and Electrode was included as a within-subject factor in the statistical analysis. In view of the uncertainty pertaining to the timing, the infant response was analyzed across eight 50-ms windows between 100 and 500 ms after stimulus onset, and Time Bin was included as a within-subject factor in the statistical analysis. After observing that all effects involving Electrode or Time Bin were insignificant, the infant MMR amplitudes were pooled across electrodes and time bins, thus reducing them to one number for each participant only, reflecting the mean MMR amplitude in a 50-ms window between 100 and 500 ms after stimulus onset, and across electrodes.

In sum, the adult MMR amplitude was the mean amplitude at Fz in one data-dependent 50-ms window determined between 150 and 250 ms after stimulus onset, and the infant MMR amplitude was the mean amplitude averaged across eight electrodes and all eight 50-ms windows between 100 and 500 ms after stimulus onset.

### 7. Comparing infant and adult MMRs: normalization

Even after minimizing methodological differences between testing infants and testing adults, it was possible that the MMR amplitudes (as computed in the previous section) still incorporated differences between the age groups that do not pertain to neural discrimination. In an attempt to filter out these residual differences, we examined whether a quantifiable relation between infant and adult MMR amplitudes could be deduced from previous literature. Because the difference between the test vowels [æ] and [ε] can be termed a difference in vowel quality, we looked for pairs of adult and infant studies in which MMRs in response to the *same* vowel quality differences were recorded. Table 1 presents the MMR amplitudes in the pairs of studies found in the literature.

**Table 1:** Adult and infant studies in which MMRs to the same vowel pairs differing in quality ([standard]–[deviant]) were recorded.

Vowel stimuli	Adults		Infants			
	Study	MMR	Study	Age	Sleep stage	MMR
**[y]–[i]**	[45]	−4.5^a^	[24]	0	quiet sleep	−1.3^b^
	[45]	−4.5^a^	[26]	3	awake	−4.0^c^
	[45]	−4.5^a^	[46]	0	quiet sleep	−1.7^d^
	[45]	−4.5^a^	[46]	3	awake	−3.1^d^
**[y]–[y/i]**	[45]	−3.3^a^	[26]	3	awake	−2.0^c^
**[e]–[ø]**	[30]	−1.6^e^	[35]	6	awake	−4.5^f^
**[e]–[o] (adults), [o]–[e] (infants)**	[30]	−2.0^e^	[47]	0	active sleep	−1.8^g^
	[30]	−2.0^e^	[47]	0	quiet sleep	−2.1^g^
**[i**:**]–[e**:**]**	[44]	−3.5^h^	[25]	0	several	+1.7^h^

The MMR amplitude (MMR; in microvolts) is listed for both the adults and the infants. For the infants, age (in months) and sleep stage are also shown.

a Amplitudes calculated from the amplitudes mentioned for the “Ignore condition” at Fz ([45]: p.202).

b Amplitude calculated from the amplitudes at F3 and F4 between 200 and 300 ms after stimulus onset.

c Amplitude at C4 (peak observed between 200 and 300 ms).

d Amplitude at F4.

e Amplitude at Fz inferred from graph ([30]: Figure 4a on page 434).

f Amplitudes at Cz inferred from graph ([35]: Figure 3 on page 353).

g Amplitudes averaged across Fp1, Fp2, C3 and C4, and across MMN (measured between 100 and 300 ms) and LDN (measured between 300 and 500 ms).

h Only the MMRs obtained in an oddball paradigm (the MMRs obtained in a multifeature paradigm are not included). At Fz in [44]. At F3 and F4 in [25].

When aiming to quantify the relation between adult and infant MMRs, the first issue to be addressed is a potential *polarity* difference, as the table shows for [i:]–[e:]. As mentioned above (Method section 6), adult MMRs are commonly negative. Infant MMRs differ in polarity across studies. In some studies they are negative (as in many studies in Table 1), in other studies positive (e.g., [25], [48]–[50]), and in still other studies both negative and positive MMR components are reported (e.g., [51]–[53]). To accommodate polarity differences between infant and adult MMRs, we consider from now on the *absolute values* of the mean MMR amplitudes in Table 1.

The second issue to be addressed in a comparison of adult and infant MMRs is the *size* of the MMR. If we collapse all MMR amplitudes listed in Table 1 per vowel (regardless of factors such as age and sleep stage) and then average over the five vowel contrasts, we obtain an adult average of 2.98 µV and an infant average of 2.54 µV. Based on these numbers, infant MMRs become comparable to adult MMRs if they are multiplied by a scaling factor of 1.18. We could be more precise and restrict ourselves to studies where the vowels are matched and where two factors that may influence the MMR amplitude, namely age [29] and sleep stage [51], are taken the same for the infants as in [4]. In that case only three comparisons between infant and adult MMR amplitudes are left in Table 1, namely those where the infants were 3 months old and were awake. The absolute MMR amplitudes in these studies were 4.0 µV [26] or 3.1 µV [46] for infants versus 4.5 µV [45] for adults, and 2.0 µV [26] for infants versus 3.3 µV [45] for adults, which would lead to a scaling factor of 1.41. Another factor that can affect the MMR amplitude is the offset-to-onset inter-stimulus interval [54]. If this inter-stimulus interval is required to be the same in the infant study as in the adult study, only one comparison mentioned in the table is left: 3.5 µV [44] vs. 1.7 µV [25]. This would yield a (too unreliable) scaling factor of 2.05.

As the scaling factors thus determined are based on a very small sample of studies, the analyses below will include a *range* of scaling factors for the infant MMR amplitudes rather than just one or two. In addition, because the polarity of the MMR in the infants in [4] was positive and a negative polarity is expected for the adults, we will multiply the adult MMR amplitudes by −1 before comparing them to the MMR amplitudes of the infants in [4].

## Results

### 1. Descriptives

#### Grand average waveforms

**Figure 2:**
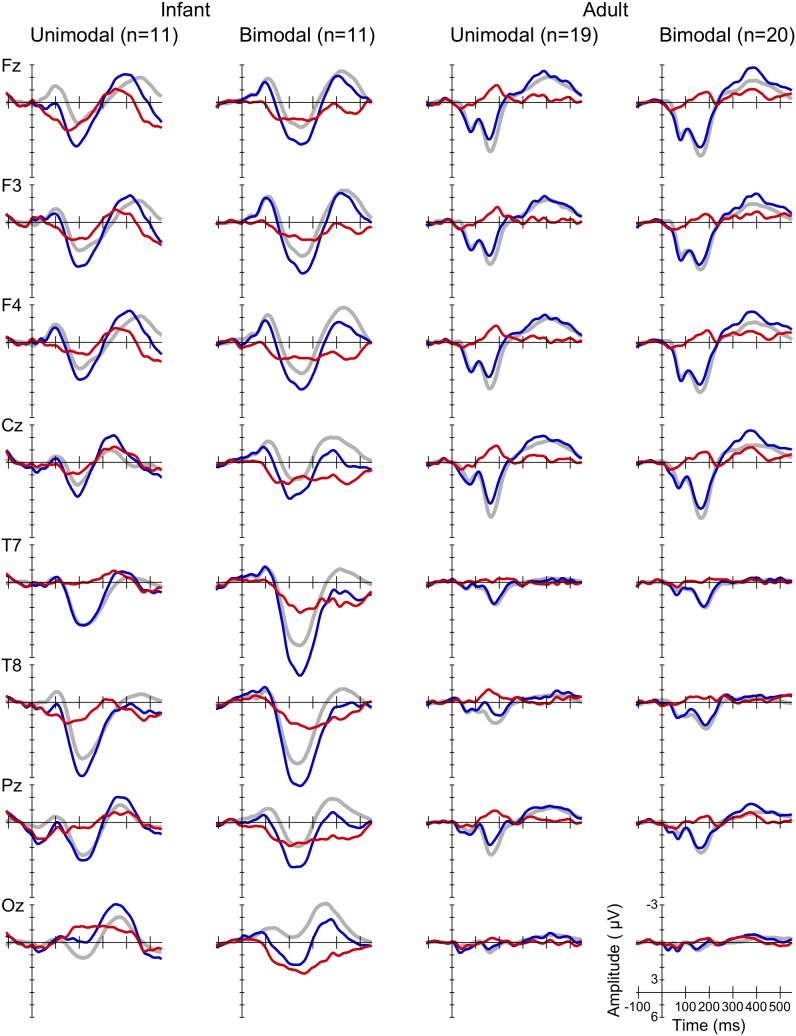
Grand average waveforms. Standard (grey, thick curves), deviant (blue, thin curves) and MMR (red, thin curves), at eight electrodes (see rows), for the unimodally and bimodally trained infants in [4] (the two columns on the left) and adults in the current study (the two columns on the right).

Figure 2 shows the grand average standard, deviant and MMR waveforms of the adults in the current study (right) and, for comparison, of the infants in [4] (left), at eight electrodes, for each Distribution Type (unimodal vs. bimodal) pooled over Standard Vowel. The figure confirms the negative polarity and the expected latency and fronto(central) scalp distribution of the adult MMN (Method section 6): the red curve, which is the MMR waveform, deviates in the negative direction (notice that negative polarities are plotted upwards) from the baseline between 150 and 250 ms, and seems to do so more at frontocentral sites then elsewhere. The figure also confirms that the infant MMR contains less pronounced peaks [55] and that its scalp distribution is less defined than in adults (e.g., [54], see also [4]). Also, in accordance with several previous studies (e.g., [25], [48]–[50]), the polarity of the infant MMR is positive.

#### Scalp distributions

**Figure 3:**
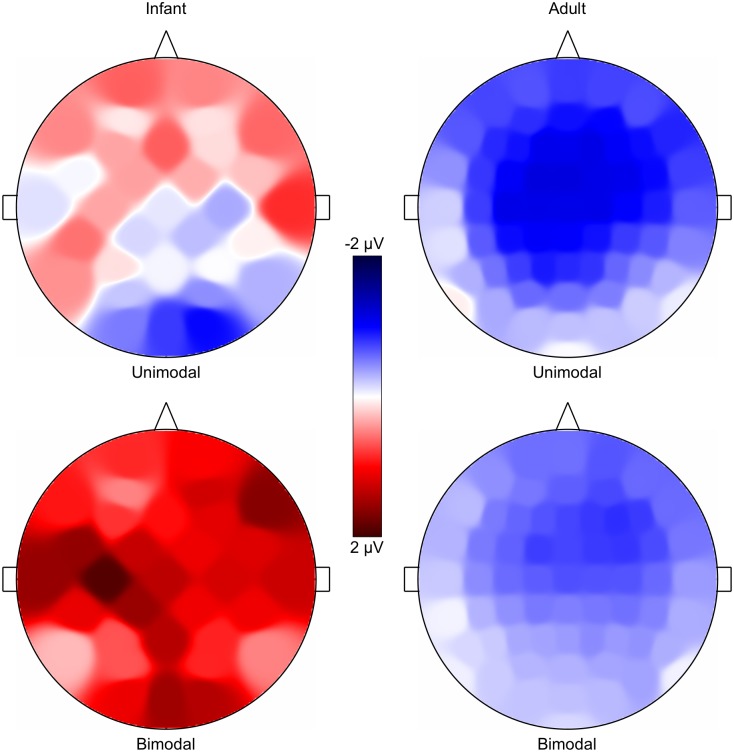
MMR scalp distributions. Unimodally (top) and bimodally (bottom) trained infants in [4] (left; 32 electrodes) and adults in the current study (right; 64 electrodes). Voltages time-averaged between 167 and 217 ms after stimulus onset for adults, between 100 and 500 ms for infants. Blue is negative, red positive, white zero.

Figure 3 depicts the scalp distributions, which were made in Praat [43], for the unimodally (top) and bimodally (bottom) trained adults in the current study (right) and, for comparison, for the infants in [4] (left). The adult distributions were measured between 167 and 217 ms after stimulus onset, i.e., in a 50-ms window around the average MMR latency (i.e., the time of the most negative voltage occurring in the grand average waveform at Fz between 150 and 250 ms), which was at 192 ms. The infant distributions were measured between 100 and 500 ms after stimulus onset (Method section 6). Just as the grand average waveforms in Figure 2, the topographies of the MMR in Figure 3 illustrate the adult negative polarity (always blue, never red) and frontocentral distribution (darkest blue at frontocentral sites). For the infants, the positive polarity (red) and less specified distribution (darkest colors are spread over the scalp) are clearest for the bimodally trained infants. The MMR was not significantly different from zero for the unimodally trained infants (details are provided in Results section 2).

#### MMR amplitudes

The MMR amplitude in the overall window where the response was expected (i.e., between 150 and 250 ms after stimulus onset; see Method 6) was significantly negative for both the bimodally trained adults (mean = −0.45 µV, 95% confidence interval [henceforth CI] = −0.95∼−0.05 µV, *t*[19] = −1.89, *p* = 0.037) and the unimodally trained adults (−0.80 µV, 95% CI = −1.39∼−0.20 µV, *t*[18] = −2.82, *p* = 0.006), thus suggesting that both groups discriminated the two test vowels to some extent.

Subsequently, for each adult participant the MMR amplitude was calculated at Fz in a 50-ms window around the MMR latency for the participant’s group (see Method 6). This group latency was 193 ms for Unimodal [æ], 196 ms for Bimodal [æ], and 189 ms for Unimodal [ε] and Bimodal [ε]. The MMR amplitudes, averaged over the participants per Distribution Type and Standard Vowel, are presented in Table 2, together with their standard deviations and confidence intervals. For comparison, the corresponding numbers of the infant MMR amplitudes (see Method 6) are also shown.

**Table 2:** Mean MMR amplitudes (in µV) for the adults in the current study and the infants in [4].

Age Group	Distribution Type	Standard Vowel	N	Mean	SD	Confidence Interval	
						Lower limit	Higher limit
**Adult**	**Unimodal**	**[ε]**	10	−1.12	0.99	−1.82	−0.41
		**[æ]**	9	−1.05	1.65	−2.31	+0.22
	**Bimodal**	**[ε]**	11	−0.35	0.86	−0.93	+0.23
		**[æ]**	9	−1.21	1.32	−2.23	−0.19
**Infant**	**Unimodal**	**[ε]**	6	−0.59	0.86	−1.71	+0.52
		**[æ]**	5	+1.21	1.23	−0.71	+3.14
	**Bimodal**	**[ε]**	5	+2.26	0.83	+0.97	+3.55
		**[æ]**	6	+0.48	0.80	−0.55	+1.50

With within-group standard deviations (SD) and 95% confidence intervals, calculated per Distribution Type and Standard Vowel.^a^

a For the infants the alpha level for the confidence intervals is 2.5% instead of 5%, because the infant study included an additional group of sleeping infants. For details see [4].

In [4], no significant difference had been observed between the *infant* MMR amplitudes at frontal, central and temporal electrodes (Fz, F3, F4, Cz, C3, C4, T7, T8). To further explore the frontocentral scalp distribution observed in the *adult* grand average waveforms and scalp topographies, we performed an analysis of variance (ANOVA) with Electrode (the same eight electrodes as for the infants) as a within-subject factor. The effect of Electrode was significant (*F* [7*ε*, 266*ε*, *ε* = 0.504] = 9.94, Greenhouse–Geisser corrected *p*<0.001). The amplitude at T7 (mean = −0.19 µV) was significantly less negative (“smaller”) than the amplitudes at all frontal and central electrodes (mean at Fz = −0.91 µV, mean at Cz = −0.90 µV, mean at F3 = −0.77 µV, mean at F4 = −0.93 µV, mean at C3 = −0.85 µV, mean at C4 = −0.84 µV; all *p*s≤0.002), and not significantly different from the amplitude at T8 (mean = −0.50 µV, *p* = 0.80). These results are in line with a predominantly frontocentral distribution of the adult MMN.

### 2. No significant effect of distributional vowel training in Dutch adults

Recall (Method section 1) that in order to test whether there was a difference between the unimodally and bimodally trained participants, while controlling for differences in the presented standard, we performed an ANOVA with the MMR amplitude at Fz as the dependent variable, and with Distribution Type (unimodal vs. bimodal) and Standard Vowel ([æ] vs. [ε]) as between-subject factors. The main and interaction effects were not significant (for Distribution Type: mean difference bimodal – unimodal = +0.30 µV, 95% CI = −0.50∼+1.10 µV, *F*<1, *p* = 0.45; for Standard Vowel: mean difference [æ]–[ε] = −0.40 µV, 95% CI = −1.19∼+0.40 µV, *F*[1, 35] = 1.02, *p* = 0.32; for the interaction: *F*[1, 35] = 1.41, *p* = 0.24). Because the effects involving Standard Vowel were not significant, the amplitude data do not show proof of any perceptual asymmetry (Method section 1). The insignificance of all effects involving Distribution Type implies that the amplitude data do not provide sound evidence that bimodally trained Dutch adult learners have a different amplitude (mean = −0.78 µV, 95% CI = −1.34∼−0.23 µV) and thus benefit differently from distributional training than unimodally trained learners (mean = −1.08 µV, 95% CI = −1.65∼−0.51 µV). For comparison, the corresponding ANOVA for the infants in [4], which also included Time Bin and Electrode as within-subject factors (see Method 6), had yielded a significant effect of Distribution Type (mean difference bimodal – unimodal = +1.06 µV, 95% CI = +0.08∼+2.04 µV, *F*[1, 18] = 7.03, *p* = 0.016), with a larger positive MMR, and thus a larger effect of distributional training, for the bimodally trained infants (mean = +1.37 µV, 95% CI = +0.68∼+2.05 µV) than for the unimodally trained infants (mean = +0.31 µV, 95% CI = −0.38∼+1.00 µV).

### 3. Smaller effectiveness of distributional training in adults than in infants

From the statistical significance of the distributional effect in infants [4] and the statistical non-significance of the effect in adults (the present paper) we cannot yet conclude that the effect is greater in infants than in adults. A valid test requires a direct comparison of the two age groups. The difference in MMR amplitude between the Bimodal and Unimodal groups (i.e., Bimodal MMR – Unimodal MMR) for the adults was +0.30 µV ( = −0.78 µV–−1.08 µV; i.e., in the unexpected direction, though non-significant), whereas that for the infants [4] was +1.06 µV ( = +1.37 µV–+0.31 µV). This age difference does not appear to be due to adults having a smaller MMR amplitude in general than infants, because the literature review in the Method section (section 7) suggested that this amplitude is probably *greater* in adults than in infants. The age difference could therefore be due to a truly smaller effect of distributional training in adults than in infants. To verify this, the current section presents a numerical comparison of the infant and adult MMR amplitudes. As determined by the literature review in the Method section (section 7), the comparison requires a normalization of the MMR amplitudes, which should include a correction for the opposite polarity of adult and infant MMRs and a scaling of the size of the MMR. To implement the normalization (or something equivalent to normalization), we multiplied each adult’s MMR amplitude by −1 to correct for the negative polarity, and we multiplied each infant’s MMR amplitude by a scaling factor to correct for the smaller size. Before applying the scaling factors estimated from the literature, which were 1.18 and 1.41 (Method section 7), we present the results for a more conservative scaling factor of 1.00 (i.e. no scaling), which is smaller than the estimates; this scaling turns the mean MMR for adults into −0.30 µV, and that for the infants into +1.06 µV, giving a difference of 1.36 µV.

#### Scaling factor of 1

**Figure 4:**
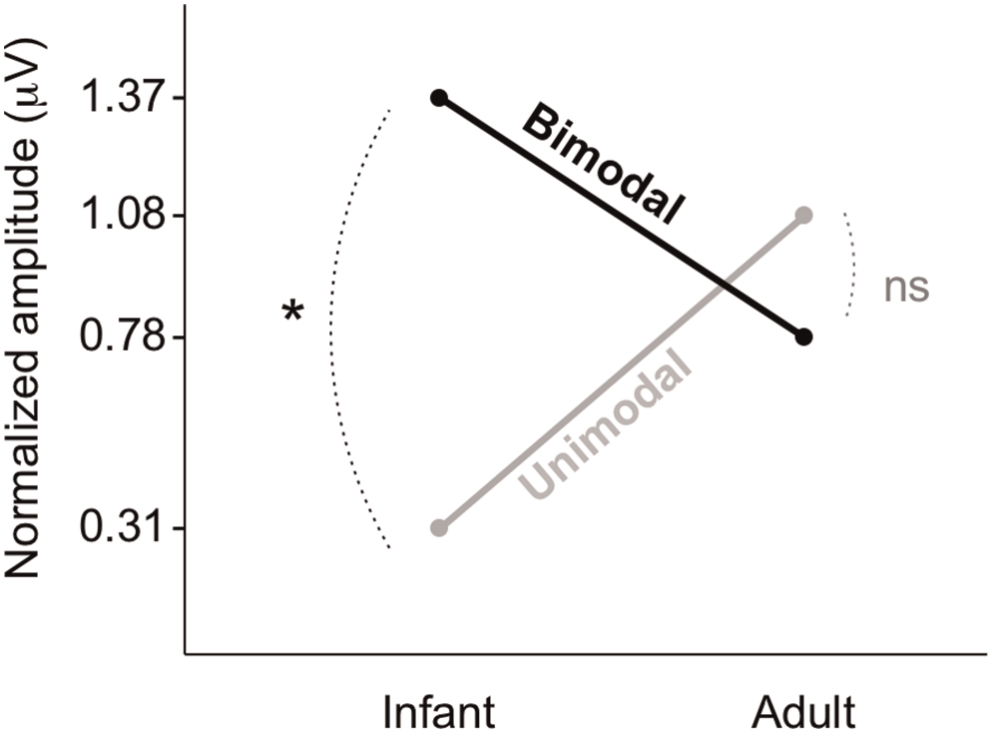
The interaction between Age Group and Distribution Type. Age group: infant, left vs. adult, right. Distribution Type: unimodal, grey vs. bimodal, black.

Using a conservative scaling factor of 1, we performed an ANOVA with the normalized MMR amplitude as the dependent variable, and Age Group (infant vs. adult), Distribution Type (unimodal vs. bimodal) and Standard Vowel ([æ] vs. [ε]) as between-subject factors (given that in [4] a strong interaction was observed between Distribution Type and Standard Vowel, Standard Vowel was included to be able to extract possible interactions with this variable). The ANOVA yielded the following normalized MMR amplitudes per Age Group and Distribution Type (as visible in Figure 4): infant unimodal 0.31 µV (CI = −0.38∼+1.00 µV), infant bimodal 1.37 µV (CI = +0.68∼+2.05 µV), adult unimodal 1.08 µV (CI = +0.56∼+1.60 µV) and adult bimodal 0.78 µV (CI = +0.27∼+1.29 µV).

Crucially, the interaction between Age Group and Distribution Type was significant (*F*[1, 53] = 5.05, *p* = 0.029). Thus, the effect of distributional training differed between infants and adults (see below). Further, the interaction between Distribution Type and Standard Vowel was significant (*F*[1, 53] = 4.85, *p* = 0.032), as well as the triple interaction between Age Group, Distribution Type and Standard Vowel (*F*[1, 53] = 13.99, *p* = 0.0005). The other interaction effect (between Age Group and Standard Vowel) and the main effects were not significant (all *p*-values >0.21).

As the number of participants was not the same in all groups, it is relevant to note that the crucial interaction between Age Group and Distribution Type did not depend much on the way the terms for the ANOVA were entered in the linear model. With “Type-III sums of squares”, the *p*-value for each main or interaction effect is calculated from a comparison between the full model (i.e. the model with all main and interaction terms) and the full model from which only this one term was dropped. This led to the above-mentioned *p*-value of 0.029 for the interaction between Age Group and Distribution Type. With “Type-I sums of squares”, the terms are entered into the linear model one by one and the *p*-value for each term depends on when the term is added. Under the constraint that the three two-way interaction terms are added after the three main terms and before the three-way interaction term, the *p*-value for the interaction between Age Group and Distribution Type depended only slightly on the order in which the two-way interactions entered into the model: it was 0.027 if this term was entered first, 0.024 if it was entered after Distribution Type × Standard Vowel but before Standard Vowel × Age Group; 0.025 if it was entered after Standard Vowel × Age Group but before Distribution Type × Standard Vowel; and 0.023 if it was entered last. By contrast, the interaction between Distribution Type and Standard Vowel was not robust to such variation. With Type-III sums of squares, the *p*-value of the interaction was as shown above (i.e., *p* = 0.032), while with Type-I sums of squares the effect was non-significant, irrespective of the chosen order of factors (i.e., the *p*-value ranged from 0.23 to 0.27). This difference in significance is due to the strong effect of the three-way interaction term: only if this triple term is present and has taken away much of the variance does the interaction between Distribution Type and Standard Vowel provide a significant improvement to the model. The robustness of the interaction of Age Group and Distribution Type, together with the lack of robustness of the interaction of Distribution Type and Standard Vowel, means that the former effect has been shown more credibly than the latter.

The observed interaction between Age Group and Distribution Type is pictured in Figure 4. The figure suggests that the difference in the normalized MMR amplitude between unimodally and bimodally trained participants was larger (i.e., more positive after normalization) for the infants than for the adults. When controlling for a possible effect of Standard Vowel, this difference is significant for the infants (mean difference normalized bimodal – unimodal = +1.06 µV, 95% CI = +0.09∼+2.03 µV), thus indicating an effect of distributional training, and not significant for the adults (mean difference normalized bimodal – unimodal = −0.30 µV, 95% CI = −1.03∼+0.43 µV). In view of the significance of the interaction between Age Group and Distribution Type, it is now possible to interpret the significant effect of distributional training for the infants as indeed being larger (i.e., +1.06–−0.30 µV = +1.36 µV, 95% CI = +0.15∼+2.57 µV) than the non-significant effect for the adults (if that effect exists at all).

#### Other scaling factors

The statistical significance of the result depended on the size of the scaling factor by which the infant MMR amplitude was multiplied. With the conservative value of 1.00 used above, the *p*-value for the interaction between Age Group and Distribution Type was 0.029 (Type-III sums of squares). With the scaling factors estimated above (Method section 7), namely 1.18 and 1.41, which express the idea that adult MMRs are bigger than infant MMRs, the *p*-value would be lowered to 0.018 and 0.010, respectively. With a scaling factor of 0.8172, which expresses the opposite assumption from that derived from the literature, namely that infants have a somewhat *larger* MMR amplitude than adults, the *p*-value would become exactly 0.05. We can conclude that for a large range of plausible scaling factors, the effect of distributional training is reliably smaller for adults than for infants.

## Discussion

The current study provides the first evidence in a direct comparison that distributional training of speech sounds is less effective in adulthood, when new languages must be mastered, than in the first months of life, when infants start acquiring native speech sounds. Specifically, an earlier study [4] showed that Dutch 2-to-3-month-old infants who are exposed to a bimodal distribution encompassing the Southern British English vowel contrast /æ/∼/ε/, have a larger MMR amplitude, and thus supposedly discriminate the two test vowels [æ] and [ε] better, than infants exposed to a unimodal distribution. The current study demonstrates that this bimodal advantage is smaller (if at all present) in Dutch adults than in Dutch infants.

The presence of a bimodal advantage in Dutch adults is uncertain, because the difference in test vowel perception between bimodally and unimodally trained adults was not significant. It may be hypothesized that this non-significance was due to a ceiling effect (i.e., top discrimination) in both groups. After all, in the Netherlands English is a compulsory subject of study in middle school and high school, and it is also a language that can be listened to easily on television and other media. However, such a ceiling effect is unlikely. The MMR amplitudes in both groups were rather small (with 95% confidence intervals close to zero), suggesting relatively poor discrimination (cf., the amplitudes in adults listed in Table 1). Moreover, it has been shown that *despite* their experience with English, Dutch adults have trouble distinguishing the English vowels that were used in the current study [6]–[9]. Similar results have also been obtained with other languages: for instance, adult native speakers of Spanish have considerable trouble in discriminating tokens of Dutch /ɑ/- and /a/, irrespective of the length of exposure to the Dutch language [56].

Notwithstanding our efforts to make a sound comparison of the effect of distributional training in infants and adults, it is clear that future research is needed to replicate our results and to confirm the feasibility of our approach. For confirming this feasibility, it will be particularly important to ascertain that infant MMRs truly reflect behavioral discrimination just as adult MMRs do (section 1 below). Relatedly, future research should aim to get a much more detailed understanding of the neural processes underlying infant and adult MMRs, so that differences between them can be explained better (section 2 below presents a tentative rough explanation for the current results).

### 1. Measuring learning in adults and infants

The comparison of the effect of distributional training in adults versus infants was based on the MMR amplitudes. Our approach featured a minimization of methodological differences between testing infants and testing adults, and a normalization of the MMR amplitudes prior to statistical analysis in order to filter out possible residual differences between adult and infant MMRs. We presented a range of feasible normalization factors to account for the scarcity of information available for estimating such a factor in the literature, and to accommodate different possibilities of calculating such a factor.

Still, an important concern in our approach remains, which, notably, also applies to other outcome measures (such as looking times) in other paradigms. This concern is that the MMR may not reflect the same processes in adults as in infants. In particular, it is important to ascertain in future research whether the infant MMR indeed reflects behavioral discrimination. This has been assumed widely on the basis of evidence in adults (for a review see [23]), but has never been verified experimentally. In this context it is noteworthy that a discrepancy between behavioral and neurophysiological measures also exists in the literature on auditory thresholds. These thresholds appear to be much higher in infants than in adults in the behavioral literature [57], but less so in research where auditory brainstem responses have been measured [58]. It has been suggested that this discrepancy occurs due to the co-existence of a mature auditory system and an immature system necessary for making efficient use of this auditory system; the discrepancy can then arise when behavioral measures tap the immature system, while neurophysiological measures tap the mature system [58], [59]. To examine whether the infant MMR truly reflects behavioral discrimination, it seems therefore important to relate behavioral measures (such as high-amplitude sucking measures for the youngest infants, and eye-tracking measures for older infants) with MMR recordings.

### 2. Top-down influence on bottom-up learning

It is not certain whether the observed smaller effect of distributional training in adults than in infants is due to a weakened distributional learning mechanism, which is generally considered to represent a purely stimulus-driven, and thus *bottom-up* learning mechanism [1], [2], or rather to strengthened *top-down* processing, or perhaps to both of these factors. Top-down processing refers to the modulation of stimulus-driven neural activity in lower-level areas (e.g., the primary auditory cortex) by higher-level linguistic representations (e.g., phonological word forms). In 2-to-3-month olds such top-down influence is lacking, because they do not have such higher-level representations yet [60]–[64].

The first scenario (i.e., a weakened bottom-up learning mechanism) matches the decline of neural plasticity in the course of childhood, which has been related to an increase in the difficulty of “learning” with age [65], and which has been included in successful computer simulations of distributional learning [1], [2]. The second scenario (i.e., strengthened top-down processing) is in accordance with the observation that distributional learning of human speech sounds can be measured in adult rats [66], thus suggesting that it is a low-level mechanism that remains in place after neural plasticity has reduced to adult levels. In this scenario, distributional learning can be observed in the rats, because, similarly to the 2-to-3-month olds, they do not have linguistic representations that could modulate lower-level neural activity.

A top-down influence of higher- on lower-level representations may already emerge after 4 to 5 months of life, as implied by research on the histological structure and development of the human auditory cortex [67]–[69]. This research shows that the six cortical layers that children and adults have, are not present from birth but develop in the first year of life and become visible in post-mortem tissue around 4 to 5 months. Crucially, the division into multiple layers seems to be a prerequisite for top-down influence from higher- to lower-level cortical areas [70]. A look at the functional organization of the layers may clarify this. Roughly, layer IV receives input from the thalamus and projects primarily to layers II and III (“supragranular layers”), which in turn target other parts of the cortex; layers V and VI (“infragranular layers”) receive input from the supragranular layers and project to the thalamus and other subcortical structures [71]. This functional division suggests that in order to make top-down influence from higher- to lower-level representations possible, the infant cortex must first develop supragranular layers, so that incoming signals can reach higher-level areas, where higher-level representations can be formed, and it must develop infragranular layers that receive top-down influence from these higher-level representations. At 4 to 5 months, rudimentary layering becomes visible in the tissue [68]. Although it is possible that some top-down influence from higher-level to lower-level cortical areas occurs before this time via layer I, which is the only layer that is clearly visible in post-mortem tissue at birth [67]–[69], the infrastructure for canonical top-down cortical influence thus emerges just before infants begin to perceive speech sounds in a language-specific way, which is from 6 months of life ([72], [3]; review in [4]). This opens up the possibility that this language-specific speech perception relies on top-down influence of higher-level speech sound representations. At the same time, neural plasticity is still high at 6 months (e.g., [73]), so that the possibility remains that the onset of language-specific speech perception (also) relies on bottom-up learning.

If in adults the distributional learning mechanism tends to be “suppressed” by top-down influence of higher-level native linguistic representations, the previous significant effects of adult distributional training might have been obtained because the experimental setting (entailing the absence of a natural language context) reduced the influence of these representations on perception. Alternatively, the way the training stimuli were presented may have attracted participants’ attention to the differences between the speech sounds in the tested contrast. If this is true, the observed effects of distributional training would be due to “attention”, which can be related to top-down processes in the brain (e.g., [74], [75]) rather than to distributional training, which is a strictly bottom-up mechanism.

In this respect it is noteworthy that for the adult Spanish learners of the Dutch vowel contrast /ɑ/~/a:/ in [20]–[22], *enhanced* bimodal training in particular seemed effective. Here the acoustic difference between the minimum and the maximum value along the presented continuum of the training distribution was made larger. From previous research in the second-language literature where other training paradigms than distributional training were used, it is known that widening the acoustic distance between presented stimuli in the training phase can draw participants’ attention to the differences between these stimuli and improve subsequent discrimination and categorization performance [76]–[78]. Thus, it is possible that the previous observations of “distributional learning” in adults were related to attention instead.

All in all, distributional learning as a mechanism for learning speech sounds seems to be weaker later in life than in infancy. The reduced prominence in adulthood may be due to fainter bottom-up learning as well as to the presence (versus the virtual absence in newborns) of higher-level linguistic representations and of a cortical infrastructure that enables top-down influence of these representations on bottom-up learning.

## References

[1] Lacerda F (1995) The perceptual-magnet effect: an emergent consequence of exemplar-based phonetic memory. In: Elenius K, Branderyd P, editors. Proceedings of the XIIIth International Congress of Phonetic Sciences (vol. 2). Stockholm: KTH and Stockholm University. pp. 140–147.

[2] Guenther FH, Gjaja MN (1996) The perceptual magnet effect as an emergent property of neural map formation. J Acoust Soc Am 100: 1111–1121. 8759964 10.1121/1.416296

[3] Werker JF, Tees RC (1984) Cross-language speech perception: evidence for perceptual reorganization during the first year of life. Infant Behav Dev 7: 49–63 [republished 2002 in Infant Behav Dev 25: 121–133].

[4] Wanrooij K, Boersma P, Van Zuijen TL (2014) Fast phonetic learning occurs already in 2-to-3-month old infants: an ERP study. Front Psychol (Language Sciences) 5, article 77, 12 pages. doi:10.3389/fpsyg.2014.00077. 10.3389/fpsyg.2014.00077 PMC3933791 24701203

[5] Hawkins S, Midgley J (2005) Formant frequencies of RP monophthongs in four age groups of speakers. J Int Phon Assoc 35(2): 183–199.

[6] Schouten MEH (1975) Native-language interference in the perception of second-language vowels: an investigation of certain aspects of the acquisition of a second language. Doctoral dissertation. Utrecht University.

[7] Weber A, Cutler A (2004) Lexical competition in non-native spoken-word recognition. J of Mem Lang 50: 1–25.

[8] Broersma M (2005) Perception of familiar contrasts in unfamiliar positions. J Acoust Soc Am 117: 3890–3901. 16018491 10.1121/1.1906060

[9] Escudero P, Hayes-Harb R, Mitterer H (2008) Novel second-language words and asymmetric lexical access. J Phon 36: 345–360.

[10] Adank P, Van Hout R, Smits R (2004) An acoustic description of the vowels of Northern and Southern standard Dutch. J Acoust Soc Am 116: 1729–1738. 15478440 10.1121/1.1779271

[11] Van Leussen J-W, Williams D, Escudero P (2011) A comparison of Dutch steady-state vowels: contextual effects and a comparison with previous studies. In: Lee W, Zee E., editors. Proceedings of the 17th International Congress of Phonetic Sciences. Hong Kong. pp. 1194–1197.

[12] Maye J, Werker JF, Gerken LA (2002) Infant sensitivity to distributional information can affect phonetic discrimination. Cognition 82(3): B101–B111. 11747867 10.1016/s0010-0277(01)00157-3

[13] Maye J, Weiss D, Aslin R (2008) Statistical phonetic learning in infants: facilitation and feature generalization. Dev Sci 11(1): 122–134. 18171374 10.1111/j.1467-7687.2007.00653.x

[14] Yoshida KA, Pons F, Maye J, Werker JF (2010) Distributional phonetic learning at 10 months of age. Infancy 15(4): 420–433. 10.1111/j.1532-7078.2009.00024.x 32693519

[15] Capel DJH, De Bree EH, De Klerk MA, Kerkhoff AO, Wijnen FNK (2011) Distributional cues affect phonetic discrimination in Dutch infants. In: Zonneveld W, Quené H, Heeren W, editors. Sound and sounds. Studies presented to M.E.H. (Bert) Schouten on the occasion of his 65th birthday. Utrecht: UiL-OTS. pp. 33–43.

[16] Maye J, Gerken LA (2000) Learning phonemes without minimal pairs. In: Howell SC, Fish SA, Keith-Lucas T, editors. BUCLD 24 Proceedings. Somerville, MA: Cascadilla Press. pp. 522–533.

[17] Maye J, Gerken LA (2001) Learning phonemes: how far can the input take us? In: Do AH-J, Domínguez L, Johansen A, editors. BUCLD 25 Proceedings. Somerville, MA: Cascadilla Press. pp. 480–490.

[18] Gulian M, Escudero P, Boersma P (2007) Supervision hampers distributional learning of vowel contrasts. Proc Int Congr Phon Sci (Saarbrücken, Germany, August 6–10): 1893–1896.

[19] Hayes-Harb R (2007) Lexical and statistical evidence in the acquisition of second language phonemes. Second Lang Res 23(1): 65–94.

[20] Escudero P, Benders T, Wanrooij K (2011) Enhanced bimodal distributions facilitate the learning of second language vowels. J Acoust Soc Am 130(4): EL206–EL212. 21974493 10.1121/1.3629144

[21] Wanrooij K, Escudero P, Raijmakers MEJ (2013) What do listeners learn from exposure to a vowel distribution? An analysis of listening strategies in distributional learning. J Phon 41: 307–319 10.1016/j.wocn.2013.03.005

[22] Wanrooij K, Boersma P (2013) Distributional training of speech sounds can be done with continuous distributions. J Acoust Soc Am 133(5): EL398–EL404. 23656100 10.1121/1.4798618

[23] Näätänen R, Paavilainen P, Rinne T, Alho K (2007) The mismatch negativity (MMN) in basic research of central auditory processing: a review. Clin Neurophysiol 118: 2544–2590. 17931964 10.1016/j.clinph.2007.04.026

[24] Cheour-Luhtanen M, Alho K, Kujala T, Sainio K, Reinikainen K, Renlund M, Aaltonen O, Eerola O, Näätänen R (1995) Mismatch negativity indicates vowel discrimination in newborns. Hear Res 82: 53–58. 7744713 10.1016/0378-5955(94)00164-l

[25] Partanen E, Pakarinen S, Kujala T, Huotilainen M (2013) Infants’ brain responses for speech sound changes in fast multifeature MMN paradigm. Clin Neurophysiol 124: 1578–1585 10.1016/j.clinph.2013.02.014 23523115

[26] Cheour M, Alho K, Sainio K, Reinikainen K, Renlund M, Aaltonen O, Eerola O, Näätänen R (1997) The mismatch negativity to changes in speech sounds at the age of three months. Dev Neuropsychol 13(2): 167–174.

[27] Van Zuijen TL, Plakas A, Maassen BA, Maurits NM, Van der Leij A (2013) Infant ERPs separate children at risk of dyslexia who become good readers from those who become poor readers. Dev Sci 16(4): 554–563. 23786473 10.1111/desc.12049

[28] Kraus N, Burton Koch D, McGee T, Nicol TG, Cunningham J (1999) Speech-sound discrimination in school-age children: psychophysical and neurophysiologic measures. J Speech Lang Hear Res 42: 1042–1060. 10515504 10.1044/jslhr.4205.1042

[29] Shafer VL, Yu YH, Datta H (2011) The development of English vowel perception in monolingual and bilingual infants: neurophysiological correlates. J Phon 39: 527–545. 22046059 10.1016/j.wocn.2010.11.010 PMC3201800

[30] Näätänen R, Lehtokoski A, Lennes M, Cheour M, Huotilainen M, et al (1997) Language-specific phoneme representations revealed by electric and magnetic brain responses. Nature 385: 432–434. 9009189 10.1038/385432a0

[31] Winkler I, Lehtokoski A, Alku P, Vainio M, Czigler I, et al (1999) Pre-attentive detection of vowel contrasts utilizes both phonetic and auditory memory representations. Cogn Brain Res 7: 357–369. 10.1016/s0926-6410(98)00039-1 9838192

[32] Pang EW, Edmonds GE, Desjardins R, Khan SC, Trainor LJ, et al (1998) Mismatch negativity to speech stimuli in 8-month-old infants and adults. Int J Psychophysiol 29: 227–236. 9664230 10.1016/s0167-8760(98)00018-x

[33] Näätänen R (1992) Attention and brain function. Hillsdale, NJ: Lawrence Erlbaum Associates.

[34] Aaltonen O, Eerola O, Hellström Å, Uusipaikka E, Lang AH (1997) Perceptual magnet effect in the light of behavioral and psychophysiological data. J Acoust Soc Am 101(2): 1090–1105. 9035400 10.1121/1.418031

[35] Cheour M, Čeponiené R, Lehtokoski A, Luuk A, Allik J, et al (1998) Development of language-specific phoneme representations in the infant brain. Nat Neurosci 1(5): 351–353. 10196522 10.1038/1561

[36] Näätänen R, Winkler I (1999) The concept of auditory stimulus representation in cognitive neuroscience. Psychol Bull 125(6): 826–859. 10589304 10.1037/0033-2909.125.6.826

[37] Näätänen R, Gaillard AWK, Mäntysalo S (1978) Early selective-attention effect on evoked potential reinterpreted. Acta Psychol 42: 313–329. 10.1016/0001-6918(78)90006-9 685709

[38] Schröger E (1997) On the detection of auditory deviations: a pre-attentive activation model. Psychophysiology 34: 245–257. 9175439 10.1111/j.1469-8986.1997.tb02395.x

[39] Keuroghlian AS, Knudsen EI (2007) Adaptive auditory plasticity in developing and adult animals. Prog Neurobiol 82(3): 109–121. 17493738 10.1016/j.pneurobio.2007.03.005

[40] Polka L, Bohn O-S (1996) A cross-language comparison of vowel perception in English-learning and German-learning infants. J Acoust Soc Am 100(1): 577–592. 8675849 10.1121/1.415884

[41] Polka L, Bohn O-S (2003) Asymmetries in vowel perception. Speech Commun 41: 221–231.

[42] Lahiri A, Reetz H (2010) Distinctive features: phonological underspecification in representation and processing. J Phon 38: 44–59 10.1016/j.wocn.2010.01.002

[43] Boersma P, Weenink D (2014) Praat: Doing phonetics by computer. Computer program. Available: http://www.praat.org. Accessed 2010–2014.

[44] Pakarinen S, Lovio R, Huotilainen M, Alku P, Näätänen R, et al (2009) Fast multifeature paradigm for recording several mismatch negativities (MMNs) to phonetic and acoustic changes in speech sounds. Biol Psychol 82: 219–226. 19646504 10.1016/j.biopsycho.2009.07.008

[45] Aaltonen O, Niemi P, Nyrke T, Tuhkanen M (1987) Event-related brain potentials and the perception of a phonetic continuum. Biol Psychol 24: 197–207. 3663795 10.1016/0301-0511(87)90002-0

[46] Cheour M, Alho K, Čeponiené R, Reinikainen K, Sainio K, et al (1998) Maturation of mismatch negativity in infants. Int J Psychophysiol 29: 217–226. 9664229 10.1016/s0167-8760(98)00017-8

[47] Martynova O, Kirjavainen J, Cheour M (2003) Mismatch negativity and late discriminative negativity in sleeping human newborns. Neurosci Lett 340: 75–78. 12668240 10.1016/s0304-3940(02)01401-5

[48] Dehaene-Lambertz G (2000) Cerebral specialization for speech and non-speech stimuli in infants. J Cogn Neurosci 12(3): 449–460. 10931771 10.1162/089892900562264

[49] Carral V, Huotilainen M, Ruusuvirta T, Fellman V, Näätänen R, et al (2005) A kind of auditory ‘primitive intelligence’ already present at birth. Eur J Neurosci 21: 3201–3204. 15978029 10.1111/j.1460-9568.2005.04144.x

[50] Dehaene-Lambertz G, Baillet S (1998) A phonological representation in the infant brain. Neuroreport 9(8): 1885–1888. 9665620 10.1097/00001756-199806010-00040

[51] Friedrich M, Weber C, Friederici AD (2004) Electrophysiological evidence for delayed mismatch response in infants at-risk for specific language impairment. Psychophysiology 41: 772–782. 15318883 10.1111/j.1469-8986.2004.00202.x

[52] Morr ML, Shafer VL, Kreuzer JA, Kurtzberg D (2002) Maturation of Mismatch Negativity in typically developing infants and preschool children. Ear Hear 23(2): 118–136. 11951848 10.1097/00003446-200204000-00005

[53] Friederici AD, Friedrich M, Weber C (2002) Neural manifestation of cognitive and precognitive mismatch detection in early infancy. Neuroreport 13(10): 1251–1254. 12151780 10.1097/00001756-200207190-00006

[54] Cheour M, Čeponiené R, Leppänen P, Alho K, Kujala T, et al (2002) The auditory sensory memory trace decays rapidly in newborns. Scand J Psychol 43: 33–39. 11885758 10.1111/1467-9450.00266

[55] Kushnerenko E, Čeponiené R, Balan P, Fellman V, Huotilainen M, et al (2002) Maturation of the auditory event-related potentials during the first year of life. Neuroreport 13(1): 47–51. 11924892 10.1097/00001756-200201210-00014

[56] Escudero P, Wanrooij K (2010) The effect of L1 orthography on non-native vowel perception. Lang Speech 53(3): 343–365. 21033651 10.1177/0023830910371447

[57] Werner LA, Gillenwater JM (1990) Pure-tone sensitivity of 2- to 5-week old infants. Infant Behav Dev 13: 355–375.

[58] Werner LA (1996) The development of auditory behavior (or What the anatomists and physiologists have to explain). Ear Hear 17(5): 438–446. 8909892 10.1097/00003446-199610000-00010

[59] Kushnerenko E (2003) Maturation of the cortical auditory event-related brain potentials in infancy. Doctoral dissertation. University of Helsinki.

[60] Jusczyk PW, Aslin RN (1995) Infants’ detection of the sound patterns of words in fluent speech. Cogn Psychol 29: 1–23. 7641524 10.1006/cogp.1995.1010

[61] Hallé PA, De Boysson-Bardies B (1996) The format of representation of recognized words in infants’ early receptive lexicon. Infant Behav Dev 19: 463–481.

[62] Jusczyk PW, Hohne EA (1997) Infants’ memory for spoken words. Science 277: 1984–1986. 9302291 10.1126/science.277.5334.1984

[63] Fikkert P (2010) Developing representations and the emergence of phonology: evidence from perception and production. Lab Phonol 10: 227–260.

[64] Bergelson E, Swingley D (2012) At 6–9 months, human infants know the meanings of many common nouns. Proc Natl Acad Sci USA 109: 3253–3258. 22331874 10.1073/pnas.1113380109 PMC3295309

[65] Kral A, Hartmann R, Tillein J, Heid S, Klinke R (2001) Delayed maturation and sensitive periods in the auditory cortex. Audiol Neurootol 6(6): 346–362. 11847463 10.1159/000046845

[66] Pons F (2006) The effects of distributional learning on rats’ sensitivity to phonetic information. J Exp Psychol Anim Behav Process 32: 97–101. 16435970 10.1037/0097-7403.32.1.97

[67] Moore JK, Guan Y-L (2001) Cytoarchitectural and axonal maturation in human auditory cortex. J Assoc Res Otolaryngol 2(4): 297–311. 11833605 10.1007/s101620010052 PMC3201070

[68] Moore JK (2002) Maturation of human auditory cortex: implications for speech perception. Ann Otol Rhinol Laryngol 111: 7–10. 10.1177/00034894021110s502 12018354

[69] Moore JK, Linthicum FH (2007) The human auditory system: a timeline of development. Int J Audiol 46(9): 460–478. 17828663 10.1080/14992020701383019

[70] Kral A, Eggermont JJ (2007) What’s to lose and what’s to learn: development under auditory deprivation, cochlear implants and limits of cortical plasticity. Brain Res Rev 56: 259–269. 17950463 10.1016/j.brainresrev.2007.07.021

[71] Bastos AM, Usrey WM, Adams RA, Mangun GR, Fries P, et al (2012) Canonical microcircuits for predictive coding. Neuron 76: 695–711. 23177956 10.1016/j.neuron.2012.10.038 PMC3777738

[72] Kuhl PK, Williams KA, Lacerda F, Stevens KN, Lindblom B (1992) Linguistic experience alters phonetic perception in infants by 6 months of age. Science 255(5044): 606–608. 1736364 10.1126/science.1736364

[73] Huttenlocher PR, Dabholkar AS (1997) Regional differences in synaptogenesis in human cerebral cortex. J Comp Neurol 387: 167–178. 9336221 10.1002/(sici)1096-9861(19971020)387:2<167::aid-cne1>3.0.co;2-z

[74] Posner MI, Petersen SE (1990) The attention system of the human brain. Annu Rev Neurosci 13: 25–42. 2183676 10.1146/annurev.ne.13.030190.000325

[75] Roelfsema PR (2011) Attention – voluntary control of brain cells. Science 332: 1512–1513. 21700861 10.1126/science.1208564

[76] Jamieson DG, Morosan DE (1986) Training non-native speech contrasts in adults: acquisition of the English /ð/ - /θ/ contrast by francophones. Percept Psychophys 40(4): 205–215. 3580034 10.3758/bf03211500

[77] Iverson P, Hazan V, Bannister K (2005) Phonetic training with acoustic cue manipulations: a comparison of methods for teaching English /r/-/l/ to Japanese adults. J Acoust Soc Am 118: 3267–3278. 16334698 10.1121/1.2062307

[78] Kondaurova M, Francis A (2010) The role of selective attention in the acquisition of English tense and lax vowels by native Spanish listeners: comparison of three training methods. J Phon 38: 569–587. 21499531 10.1016/j.wocn.2010.08.003 PMC3076995

